# The Identification of a SIRT6 Activator from Brown Algae *Fucus distichus*

**DOI:** 10.3390/md15060190

**Published:** 2017-06-21

**Authors:** Minna K. Rahnasto-Rilla, Padraig McLoughlin, Tomasz Kulikowicz, Maire Doyle, Vilhelm A. Bohr, Maija Lahtela-Kakkonen, Luigi Ferrucci, Maria Hayes, Ruin Moaddel

**Affiliations:** 1 Biomedical Research Center, National Institute on Aging, National Institutes of Health, 251 Bayview Boulevard, Baltimore, MD 21224, USA; minna.rahnasto@uef.fi (M.K.R.-R.); kulikowiczt@grc.nia.nih.gov (T.K.); maire.doyle@nih.gov (M.D.); BohrV@grc.nia.nih.gov (V.A.B.); FerrucciLu@grc.nia.nih.gov (L.F.); 2 School of Pharmacy, University of Eastern Finland, Kuopio FI-70210, Finland; maija.lahtela-kakkonen@uef.fi; 3 Food Biosciences Department, Teagasc Food Research Centre, Ashtown, Dublin 15, Ireland; Padraig.McLoughlin@teagasc.ie (P.M.); Maria.Hayes@teagasc.ie (M.H.)

**Keywords:** sirtuin activators, fucoidan, brown seaweed

## Abstract

Brown seaweeds contain many bioactive compounds, including polyphenols, polysaccharides, fucosterol, and fucoxantin. These compounds have several biological activities, including anti-inflammatory, hepatoprotective, anti-tumor, anti-hypertensive, and anti-diabetic activity, although in most cases their mechanisms of action are not understood. In this study, extracts generated from five brown algae (*Fucus dichitus*, *Fucus vesiculosus* (Linnaeus), *Cytoseira tamariscofolia*, *Cytoseira nodacaulis*, *Alaria esculenta*) were tested for their ability to activate SIRT6 resulting in H3K9 deacetylation. Three of the five macroalgal extracts caused a significant increase of H3K9 deacetylation, and the effect was most pronounced for *F. dichitus*. The compound responsible for this in vitro activity was identified by mass spectrometry as fucoidan.

## 1. Introduction

SIRT6 is an NAD^+^-dependent histone deacetylase (HDACs EC 3.5.1.98) that functions as a regulator of many cellular processes, is evolutionarily conserved, and exists in a variety of organisms from eukaryotes to humans [1,2]. SIRT6 controls healthy ageing by regulating genomic stability, oxidative stress, and glucose metabolism, and it is considered a promising target for age-associated diseases such as chronic inflammation, diseases associated with metabolic syndrome, obesity, and insulin resistant type-2 diabetes [3,4,5,6,7,8]. In a recent study, it was demonstrated that SIRT6 inhibition could improve glycemia in a mouse model of type 2 diabetes [9]. Also, it was shown that SIRT6-deficient mice have a premature aging phenotype with a shortened lifespan, while the overexpression of SIRT6 prolongs the lifespan in male mice and prevents diet-induced obesity [4,10]. SIRT6 activity affects the development of several cancer subtypes, but it is still unclear whether it is a tumor suppressor or promoter, or both [2,11].

Some compounds that enhance SIRT6 activity have been identified, including fatty acids and endogenous fatty acid ethanolamides [12,13]. Phenolic compounds, such as quercetin and luteolin, also enhance SIRT6 deacetylation activity, albeit at very high concentrations [13].

Brown macroalgal species [14,15,16], specifically *Fucaceae* and *Cystoseira*, are rich in molecules that exert a large range of biological activities including phenolic compounds such as phlorotannins, and polysaccharides, such as laminarans and fucoidans. Phlorotannins protect cells against Ultraviolet (UV)-B-induced DNA modifications by inducing the nucleotide excision repair (NER) pathway of DNA repair [17]. Fucoidans are a complex heterogeneous group of sulfated polysaccharides composed of l-fucose and sulphate ester groups with minor amounts of monosaccharides that have robust anti-inflammatory and anti-proliferative effects [18,19].

Considering the elicited physiological actions of brown seaweeds and their overlap with the reported bioactivity of SIRT6, the aim of this work was to screen brown seaweed species for novel SIRT6 modulators as potential candidates that can be used in the prevention of age-associated diseases and metabolic syndrome associated disorders, including cancer, obesity, and insulin-resistant diabetes.

We used an accelerated solvent extraction (ASE^®^) method with acetone:water (70:30 *v*/*v*) [15,19] as an extraction solvent to generate phlorotannin- and fucoidan-rich extracts from five species of brown macroalgae, namely *Fucus distichus*, *Fucus vesiculous*, *Cytoseira tamariscofolia*, *Cytoseira nodacaulis,* and *Alaria esculenta*. The generated extracts were tested for their ability to deacetylate H3K9, a proxy measure of SIRT6 activity. Three of the five macroalgal extracts significantly enhanced SIRT6 activity, and the effect was most pronounced for *F. distichus*. Herein, we identify fucoidan as the compound responsible for SIRT6 activation from *F. distichus* extract using liquid chromatography and mass spectrometry.

## 2. Results

### 2.1. Screening of Brown Algae

**Figure 1:**
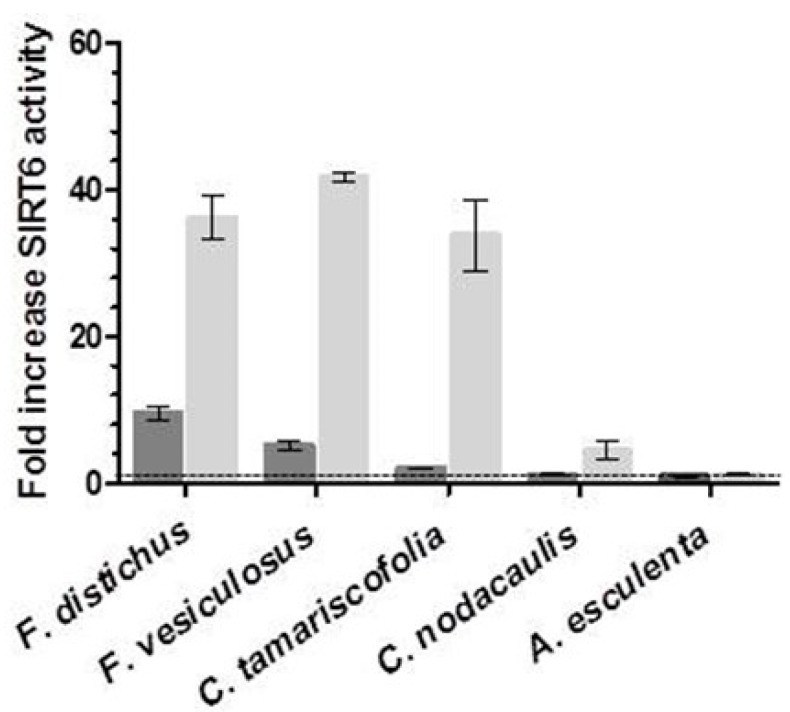
SIRT6 deacetylation activity in the presence of five species of brown algae extracts. The change of SIRT6 deacetylation in the presence of 0.5 mg/mL (grey) and 1.0 mg/mL (light grey) extracts is compared to controls with 500 µM NAD^+^ and 40 µM H3K9Ac with 30 min of incubation time. The data are presented as means ± SD, *n* = 3.

In this study, we used a previously developed HPLC deacetylation assay that estimates SIRT6 activity by measuring changes in the level of deacetylated peptide H3K9, over the substrate (H3K9Ac) [13,20], to determine SIRT6 activity in complex matrices. Five species of brown algae were tested for SIRT6 modulating activity at two concentrations (Figure 1). Of these, the *A. esculenta* ASE^®^ extract had no activity, while *C. nodacaulis* displayed SIRT6 stimulating activity, with a ~five-fold increase at 1 mg/mL compared to control. The ASE^®^ extracts from *F. distichus*, *F. vesiculosus* (Linnaeus), and *C. tamariscofolia* all displayed an approximate ~35-fold increase in SIRT6 activity when assayed at a concentration of 1 mg/mL. While the stimulation of SIRT6 activity was dose-dependent for all species tested, *F. distichus* displayed the strongest activity at 0.5 mg/mL with a ~10-fold increase. As a result, *F. distichus* was studied further.

### 2.2. Separation of F. distichus

**Figure 2:**
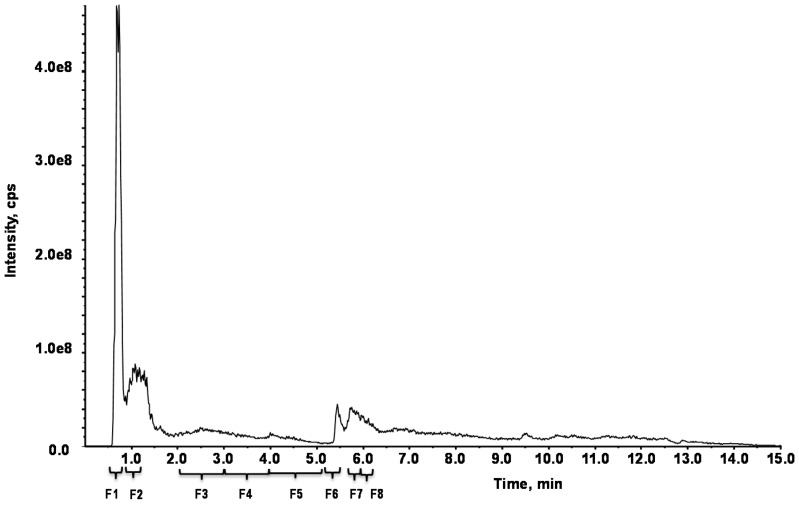
HPLC chromatogram of *F. distichus* and its separation into eight fractions using Zorbax Eclipse XDB-C18 column (4.6 mm × 50 mm, 1.8 µm). The collected fractions: F1 = 0.4–0.5 min; F7 = 5.4–5.5 min.

**Figure 3:**
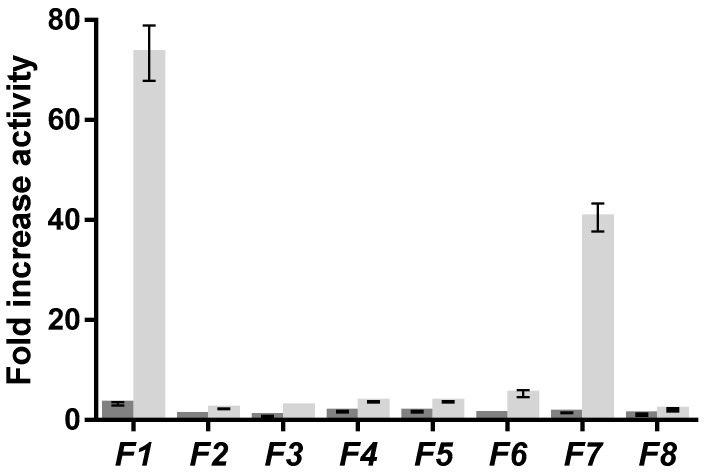
SIRT6 deacetylation activity (fold activity relative to control) of fractions (F1–F8) from *F. distichus* in the presence of 0.5 mg/mL (grey) and 1.0 mg/mL (light grey) fractions (F1–F8) with 500 µM NAD^+^ and 40 µM H3K9Ac with 30 min of incubation time. The data are presented as means ± SD, *n* = 3.

*F. distichus* was separated into eight sub-fractions (F1–F8) using an XDB-C18 column (Zorbax Eclipse) guided by the SIRT6 H3K9Ac deacetylation HPLC-based assay (Figure 2). Of the eight sub-fractions, moderate activity was observed for F3–F6, and F8 (Figure 3), with a ~3-fold increase in SIRT6 activity. Interestingly, F1 and F7 were the most active, with a ~70-fold and ~40-fold increase, respectively, in SIRT6 activity, at 1 mg/mL. Due to the increased activity observed in F1, the HPLC method was scaled up for the collection of more active fractions with semi-preparative HPLC-PDA (Supplementary Figure S1) using an Eclipse XDB-C8 (9.4 mm × 250 mm, 5 µm). Five different fractions (F1–F5) were collected and the resulting F1 fraction was further purified using a Zorbax Eclipse XDB-C18 column (4.6 mm × 50 mm, 1.8 µm), resulting in a single peak (Supplementary Figure S2).

### 2.3. Identification of Fucoidan

**Figure 4:**
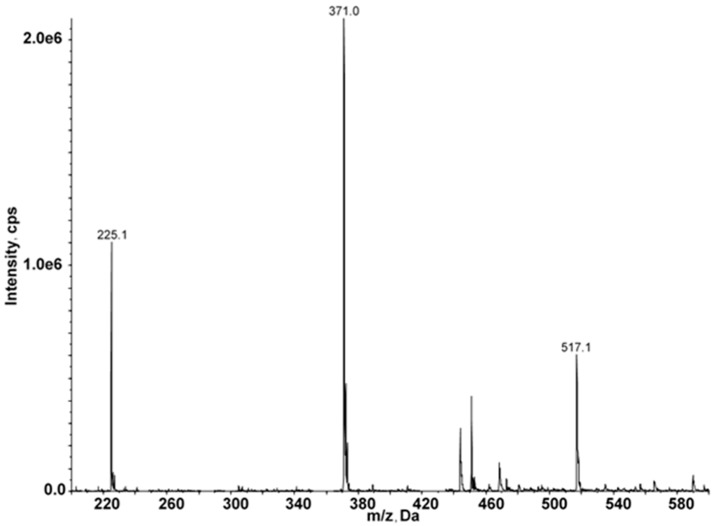
HPLC–MS analysis of subfraction F1 in negative ionization mode with a scan range of *m*/*z* 150–600.

**Figure 5:**
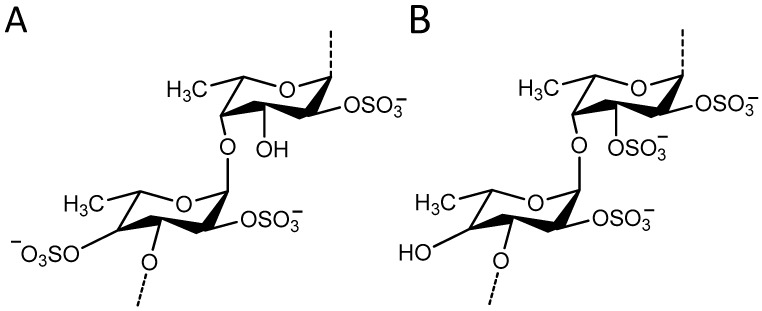
Reported structural elements for fucoidan isolated from the brown seaweeds (**A**) *F. distichus*, (**B**) *F. vesiculosus*. Modified from [24,25].

The resulting sub-fraction was characterized by mass spectrometry (Figure 4), and identified as fucoidan, a sulfated polysaccharide present in brown algae, by comparison to the reported mass spectra of isolated fucoidan from *Sargassum* genus algae [21,22]. The seaweed fucoidans are heterogenic mixtures of structurally related polysaccharides consisting of carbohydrate units (l-fucopyranose and non-fucose ones) and non-carbohydrate substituents (mainly sulfate and acetyl groups). The precise determination of their structures with the exact location of structural elements is complex. The polysaccharide backbones of fucoidans are organized in repeating (1→3)-linked or alternatively (1→3)- and (1→4)-linked α-l-fucopyranose residues [23,24]. The backbone of the fucoidan from *F. distichus* is built up mainly of the repeating A units (Figure 5), whereas fucoidans from *F. vesiculosus* are formed mainly of B units [23,24,25].

**Figure 6:**
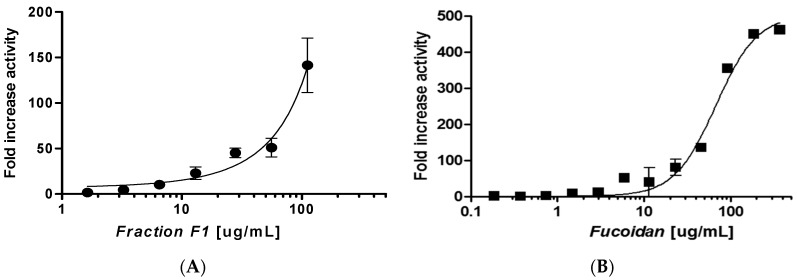
(**A**) Dose-response curve of the extract sub-fraction of F1 (●) in the presence of 500 µM NAD^+^ and 40 µM H3K9Ac with 30 min of incubation time. The data are presented as means ± SD, *n* = 3; (**B**) Dose-response curve of fucoidan (■) on SIRT6 deacetylation activity in the presence of 500 µM NAD^+^ and 40 µM H3K9Ac with 30 min of incubation time. The data are presented as means ± SD, *n* = 3.

While the employed extraction process aimed to isolate phlorotannins, the presence of fucoidan in the extracts is not unexpected, as it has been previously reported that between 0.26% and 7.0% dry weight of the algal biomass of *F. distichus* consists of fucoidan [24]. Furthermore, Béress [26] previously reported the extraction of both polyphenols and polymers including fucoidan using water-based solvent systems. It is difficult to separate polyphenols and carbohydrates based on the differential solubility of these algal components. Pantankar previously reported that fucoidan is soluble in acetone:water [27], and this is also a well-known extraction solvent for phlorotannins. A dose-response curve of a sub-fraction of F1 was carried out, and the results demonstrated a significant increase in SIRT6 activation with a ~140-fold increase observed at a 100 μg/mL concentration (Figure 6A). However, due to the limitations in the amount of *F. distichus* available, we were unable to obtain a full dose-response curve for the sub-fraction of F1. Fucoidan (>95% pure) was purchased (isolated from *F. vesiculosus*) and a full dose-response curve was carried out. The dose-response curve obtained was very similar (Figure 6B) to that obtained for F1, with a ~355-fold increase in the activity observed at 100 μg/mL.

### 2.4. Western Blot Analysis

**Figure 7:**
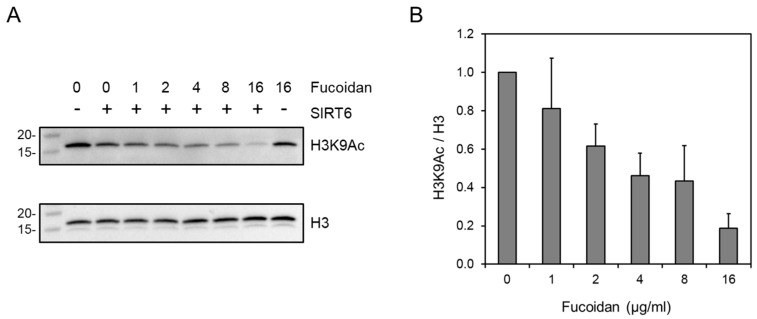
Western blot method for the in vitro SIRT6 deacetylation assay. Serially diluted concentrations of a SIRT6 stimulator (1–16 µg/mL) were incubated for 30 min at 37 °C in the presence of 1 μg/well of a purified recombinant GST-SIRT6 protein, 2 μg purified whole chicken core histones with 500 μM NAD^+^ in 25 mM Tris-HCl, pH 8.0. (**A**) Acetylation level was detected with anti-H3K9Ac antibody and normalized to total H3 histone. Values indicate final fucoidan concentration in µg/mL. Molecular weight markers in kDa. (**B**) Quantification of H3K9 deacetylation. Values represent the averages of three experiments; error bars indicate standard deviation.

The in vitro deacetylation activity was also determined by Western blot analysis, where 1 to 16 µg/mL of fucoidan was incubated with the core histones and the remaining levels of histone H3 acetylated on lysine 9 were determined. Using this technique, it was found that fucoidan activated SIRT6 deacetylation activity in a dose-dependent manner (Figure 7). Both methods demonstrate that fucoidan is an activator of SIRT6. Interestingly, it was demonstrated that higher concentrations of fucoidan (>16 µg/mL in the Western blot analysis method) resulted in the reduced activation of SIRT6 activity (data not shown). A similar observation was made in the HPLC-based assay at higher concentrations as well, indicating that fucoidan may have a dual role in SIRT6, similar to what was observed for quercetin and luteolin [13].

### 2.5. Selectivity for SIRT6

In order to determine whether fucoidan was selective for SIRT6, in vitro enzymatic assays were carried out against SIRT1, SIRT2, and SIRT3. Neither 10 μg/mL nor 100 μg/mL of fucoidan resulted in any change in the deacetylation activity of SIRT1 or SIRT3. There was a 20% reduction in SIRT2 activity at 100 μg/mL (Table 1). These results suggest that fucoidan activation of sirtuins is specific to SIRT6.

## 3. Discussion

From five species of brown algae tested against SIRT6 modulating activity, *F. distichus* displayed the most robust increase of SIRT6 deacetylation activity. As a result, the active component from *F. distichus* was identified using a guided SIRT6 H3K9Ac deacetylation HPLC-based assay as fucoidan. Due to the limitations of the starting material, commercially available fucoidan (isolated from *F. vesiculosus*) was purchased and tested for SIRT6 activity. A significant increase in deacetylation activity was observed in a dose-dependent manner using the HPLC-guided deacetylation-based assay as well Western blot analysis. The commercial fucoidan, from *F. vesiculosus*, has been reported to contain fucose (>50%), galactose (6%), glucose (20%), mannose, xylose (15%), uronic acid, glucosamine, and sulfate [28]. None of these monosaccharides introduced any SIRT6 activity at a concentration up to 300 μM (data not shown), indicating that the activity is most likely due to the sulfated fucose. Percival and Ross reported that fucoidan from *F. vesiculosus* contained 31.7% sulfation [29], while the sulfate content of commercial fucoidan from *F. vesiculosus* was estimated to be 31.2% [30,31]. Previous studies revealed that the degree of sulfation significantly influences the level of anti-angiogenic activity of fucoidans in human umbilical vein endothelial cells. For example, oversulfated fucoidan from *F. vesiculosus* with sulfate contents at 52.4% were significantly stronger at inhibiting angiogenesis than natural fucoidan from *F. vesiculosus* with sulfate contents of 31.2% [30,32]. The modulation of sirtuin activity by negatively charged sulfated polysaccharides, while novel, is not completely unexpected. For example, Tong et al. [33] reported the activation of SIRT7 deacetylation activity with negatively charged DNA. In addition, heparin and heparan sulfate proteoglycans have been reported to be potent inhibitors of HAT (Histone acetyltransferases) activity [34]. Further, fucoidan has been indicated to play a role in apoptosis [28,35,36]. In one study, it induced the cleavage of PARP (poly ADP ribose polymerase) to the 89 kDa polypeptide, suggesting that caspases were involved in the fucoidan-mediated apoptosis [37]. Furthermore, in a study in rabbits investigated fucoidan injected intramuscular (i.m.)-induced apoptosis in isolated lymphoma cell lines in vitro [28]. Similarly, SIRT6 overexpression has been demonstrated to induce apoptosis in cancer cells and not in normal cells [8]. In cancer cells, the activity was mediated via the activation of both p53 and p73 signaling cascades. Min et al. [38] reported that the treatment of HepG2 cells with fucoidan (250 and 500 μg/mL) increased the upregulation of p53 and p14, which are involved in the regulation of apoptosis, by up to two- and two and half-fold, respectively, thus inhibiting the viability of HepG2 cells. Zhang et al. (2014) [39] reported a direct interaction between SIRT6 and p53, and the activation of SIRT6 expression by intact p53, which in turn leads to an elevated association of SIRT6 with FoxO1 and the subsequent inhibition of gluconeogenesis. In addition to anti-cancer properties, fucoidans have revealed numerous other health-promoting effects, including anti-oxidative and anti-inflammatory effects. In addition to observations with SIRT6, it was demonstrated in a mouse model that low molecular weight fucoidan inhibited oxidative stress and mitochondrial dysfunction through the upregulation of the expression of SIRT3 after traumatic brain injury [40]. While these studies suggest that fucoidan could also be activating other sirtuins, the results of this study demonstrate that fucoidan, isolated from *F. distichus* and *F. vesiculosus*, is a strong stimulator of SIRT6. To our knowledge, this is the first report to identify a polysaccharide which stimulates SIRT6 deacetylation activity.

## 4. Materials and Methods

### 4.1. Materials

Acetylated histone H3 (K9) peptide (residues 1-21) (H3K9Ac) was purchased from AnaSpec Incorporation (Fremont, CA, USA). Nicotinamide adenine dinucleotide (NAD), formic acid, fucoidan from *F. vesiculosus* (F5631), and anti-rabbit HRP-conjugated secondary antibody (A0545) were ordered from Sigma Aldrich (St. Louis, MO, USA). Core histones proteins (13-107) and rabbit anti-acetyl H3K9 antibody (06-942) were ordered from Merck, EMD Millpore (Temecula, CA, USA). Rabbit anti-histone H3 antibody (9715S) was purchased from Cell Signaling Technology (Danvers, MA, USA). Novex™ WedgeWell™ 10–20% Tris-Glycine Mini Gels (12-well) (XP10202BOX) and Novex^®^ Tris-Glycine SDS Running Buffer (10×) (LC2675) were ordered from ThermoFisher Scientifics (Waltham, MA, USA).

Expression and Purification of GST-Tagged SIRT6 Protein. The human SIRT6 expression vector hSIRT6-pGEX-6P3 was kindly provided by Prof. Katrin Chua (Stanford, CA, USA). The recombinant GST (Glutathione S-transferases)-tagged SIRT6 was produced by fermentation in *Escherichia coli* BL21 (DE3)-pRARE. The production was done at +16 °C with 0.1 mM IPTG (Isopropyl β-d-1-thiogalactopyranoside), for 20 h and the soluble overexpressed protein was affinity purified on glutathione agarose (Sigma, St. Louis, MO, USA).

### 4.2. Plant Material and Extraction

#### 4.2.1. Method of Preparation

Accelerated solvent extraction (ASE^®^) was used to generate macroalgal extracts using the Dionex PLE system (ASE 200, Dionex, ThermoFisher Scientifics). Briefly, 2 g of each freeze-dried, de-fatted, and powdered macroalga sample was mixed with 4 g of silica (Merck grades, 60 A, Sigma Aldrich, Dublin, Ireland). Silica was used as an inert dispersant and the sample plus silica mixture was then packed into 22-mL extraction cells. The automated extraction method used was 70% acetone in water and a pressure and temperature of 50 °C, 1500 psi, respectively. The extraction time consisted of four cycles of 5 min. Samples were dried using a rota-evaporator at 37 °C and subsequently freeze-dried to remove water. Hexane was used to de-fat samples as previously described [41].

#### 4.2.2. Species Name

3-*Fucus distichus* (Newfoundland origin); 4-*Fucus vesiculosus* (Linnaeus) ISCG 0223; 6-*Cytoseira tamariscofolia* ISCG0283; 7-*Cytoseira nodacaulis* ISCG0070; 11-*Alaria esculenta* (Newfoundland origin); Location of Harvest and Time: 3-*Fucus distichus* (Newfoundland origin) supplied by Oceans Ltd. (St. John’s, NL, Canada) April 2010; 4-*Fucus vesiculosus* (Linnaeus) ISCG0223, Golf Course, Galway, 8 June 2011; 6-*Cytoseira tamariscofolia* ISCG0283, Finnavara, Co. Clare, 28 September 2011; 7-*Cytoseira nodacaulis* ISCG0070, Finnavara, Co. Clare, 30 March 2010; 11-*Alaria esculenta* (Newfoundland origin) kindly supplied by Dr. Anne Mathieu, Oceans Ltd., St. John’s Newfoundland. It was supplied in a freeze-dried format. All seaweeds (Irish) were rinsed and subsequently freeze-dried prior to processing and stored at −80 °C.

### 4.3. SIRT6 Deacetylation Assay

Solutions of macroalgal extracts at 0.5 and 1 mg/mL concentrations were prepared in DMSO. In this study, 0.6 µL of this solution and DMSO (control) were incubated for 30 min in the presence of 3 μg/well of SIRT6, 40 μM H3K9Ac, and 500 μM NAD^+^ in Tris Buffer (25 mM, pH 8.0) at 37 °C. Additional controls were carried out in the absence/presence of SIRT6, with or without NAD^+^ and with and without fucoidan. During the reaction, the final solvent concentration of all samples was 1% DMSO. The samples were terminated by adding 6 uL cold 10% formic acid and subsequently centrifuging for 15 min at 13.4 rpm. Dose-response effects (0.05 mg/mL to 1 mg/mL) were carried out for sub-fraction F1 from *F. distichus* and commercial fucoidan from *F. vesiculosus*.

### 4.4. HPLC Analysis

The chromatographic separation of H3K9 and acetylated H3K9 was achieved on a Zorbax Eclipse XDB-C18 column (4.6 mm × 50 mm, 1.8 µm; Agilent Technologies, Santa Clara, CA, USA) at room temperature using a Shimadzu prominence system (Shimadzu Technology, Kyoto, Japan) consisting of a CBM-20A, LC-20 AB binary pumps, an SIL-20AC-HT auto-sampler, and a DGU-20A3 degassing unit. The mobile phase consisted of water with 0.02% formic acid (elute A) and acetonitrile with 0.02% formic acid (elute B). The gradient eluent at a flow rate of 0.9 mL/min was programmed as follows: 0–2.0 min, 0% B; 2.0–10 min, 0–8% B; 10–10.10 min, 8–80% B; 10.10–12 min, 80%; 12–15 min 80–0% B; 15 min, 0% B. The total run time was 15 min and the injection volume per sample was 20 μL. The HPLC system was coupled to a 5500 QTRAP from Applied Biosystems/MDS Sciex equipped with Turbo V electrospray ionization source (TIS)^®^ (Applied Biosystems, Foster City, CA, USA). The data were acquired and analyzed using Analyst version 1.5.1 (Applied Biosystems). Positive electrospray ionization data were acquired using multiple reactions monitoring (MRM). The TIS instrumental source settings for temperature, curtain gas, ion source gas 1 (nebulizer), ion source gas 2 (turbo ion spray), entrance potential, and ion spray voltage were 550 °C, 20 psi, 60 psi, 50 psi, 10 V, and 5500 V, respectively. The TIS compound parameter settings for de-clustering potential, collision energy, and collision cell exit potential were 231, 45, and 12 V, respectively, for H3K9Ac; and were 36, 43, and 12 V, respectively, for H3K9. The standards were characterized using the following MRM ion transitions: H3K9Ac (*m*/*z* 766.339→760.690) and H3K9 (*m*/*z* 752.198→746.717).

### 4.5. HPLC Fingerprint

The chromatographic separation of *F. distichus* was achieved on a Zorbax Eclipse XDB-C18 column (4.6 mm × 50 mm, 1.8 µm; Agilent Technologies, Santa Clara, CA, USA) at room temperature using a Shimadzu prominence system (Shimadzu Technology, Kyoto, Japan) consisting of a CBM-20A, LC-20 AB binary pumps, an SIL-20AC-HT auto-sampler, and a DGU-20A3 degassing unit. The mobile phase consisted of water with 0.1% formic acid (elute A) and acetonitrile with 0.02% formic acid (elute B). The gradient eluent at a flow rate of 0.6 mL/min was programmed as follows: 0 min, 0%B; 3.0 min, 0% B; 6.0 min, 83% B; 11.0 min, 83%; 11.1 min 0% B; 15 min, 0% B. The total run time was 15 min and the injection volume per sample was 10 μl (7.5 mg/mL in 100 mM NaOH). Fractions were collected (Figure 2) between 0.4–0.5 min (F1), 1.0–1.3 min (F2), 2.0–3.0 min (F3), 3.0–4.0 min (F4), 4.0–5.0 min (F5), 5.1–5.3 min (F6), 5.4–5.5 min (F7), or 5.5–6.2 min (F8).

The HPLC system was coupled to a 5500 QTRAP from Applied Biosystems/MDS Sciex equipped with Turbo V electrospray ionization source (TIS)^®^ (Applied Biosystems, Foster City, CA, USA). The data were acquired and analyzed using Analyst version 1.5.1 (Applied Biosystems). Negative electrospray ionization data were acquired using enhanced MS (EMS) from 200 to 600 *m*/*z*. The TIS instrumental source settings for temperature, curtain gas, ion source gas 1 (nebulizer), ion source gas 2 (turbo ion spray), entrance potential, and ion spray voltage were 500 °C, 20 psi, 50 psi, 60 psi, −10 V, and −4500 V, respectively. The TIS compound parameter settings for de-clustering potential, collision energy, and collision cell exit potential were −75, −35, and 12 V, respectively.

### 4.6. SemiPREP MS

The collection of the F1 sub-fraction of *F. distichus* was achieved on an Eclipse XDB-C8 column (9.4 mm × 250 mm, 5 µm; Agilent Technologies, Santa Clara, CA, USA) at room temperature using a Shimadzu prominence system (Shimadzu Technology, Kyoto, Japan) consisting of a CBM-20A, LC-20 ADXR binary pumps, an SIL-20AC-HT auto-sampler, and a DGU-20A3R degassing unit, PDA (Photodiode array detector) SPD-M20A. The mobile phase consisted of water with 0.1% formic acid (elute A) and acetonitrile with 0.02% formic acid (elute B). The gradient eluent at a flow rate of 0.9 mL/min was programmed as follows: 0 min, 0%B; 3.0 min, 0% B; 11.0 min, 83% B; 11.1 min, 0%; 15 min, 0% B. The total run time was 15 min and the injection volume per sample was 50 μL (10 mg/mL in 100 mM NaOH). Fractions were collected (Figure 2) between 0.1–1.0 min (F1), 1.0–1.3 min (F2), 8.0–8.5 min (F3), 10.0–10.5 min (F4), or 11.0–11.4 min (F5).

The collected fractions were evaporated under nitrogen gas and dissolved two times with methanol to remove impurities. Fractions were analyzed using HPLC–MS negative ionization mode with a scan range of *m*/*z* 150–600.

### 4.7. H3K9 Western Blot Method

Fucoidan stock solution (10 mg/mL) was prepared and serially diluted in 25 mM Tris-HCl, pH 8.0. Subsequently, 1 μL of fucoidan solution or buffer control were incubated in 20 μL reaction for 30 min in the presence of 1 μg of a purified recombinant GST-SIRT6, 2 μg purified chicken core histones (Millipore, Billerica, MA, USA), and 500 μM NAD^+^ in 25 mM Tris-HCl, pH 8.0 at 37 °C. The reactions were stopped with Laemmli sample buffer and separated by SDS-PAGE using 4–15% gradient gels (Bio-Rad, Hercules, CA, USA) and transferred onto polyvinylidene difluoride (PVDF) membranes. H3K9 acetylation was detected with rabbit anti-acetyl H3K9 antibody (Millipore) followed by anti-rabbit HRP-conjugated secondary antibody. Membranes were stripped and re-probed with rabbit anti-histone H3 antibody. Chemiluminescent signal detection and image acquisition were done using SuperSignal West Femto Substrate (Thermo) and ChemiDoc XRS+ with Image Lab software (Bio-Rad).

### 4.8. In Vitro Enzymatic Assays (SIRT1-SIRT3)

The Fluor de Lys fluorescence assays were based on the method described in the BioMol product sheet (Enzo Life Sciences, Ann Arbor, MI, USA) using the BioMol KI177 substrate for SIRT1 and the KI179 substrate for SIRT2 and SIRT3. GST-SIRT1 and GST-SIRT2 were produced as described previously [42,43]. His-SIRT3 (BML-SE270) was purchased from Enzo Life Sciences. DMSO (D2650), SIRT assay buffer (HDAC assay buffer, KI143, supplemented with 1 mg/mL BSA, A3803), and NAD^+^ (N6522) were from Sigma. Fluor de Lys developer (KI176) and nicotinamide (BKI 283) were ordered form BioMol.

Briefly, the reaction mixture including acetylated peptide substrate (0.7 Km: 58 µM for SIRT1 [44], 198 µM for SIRT2 [44], and 32 µM for SIRT3), NAD^+^ (0.9 Km: 558 µM for SIRT1, 547 µM for SIRT2, and 2 mM for SIRT3), and DMSO/compounds in DMSO (2.5 μL in 50 μL total reaction volume) were preincubated for 5 min at room temperature. The reaction was started by adding the enzyme following incubation for 1 h at 37 °C. After that, the developer and nicotinamide (2 mM in HDAC assay buffer giving total volume of 50 μL) were added and the incubation was continued for 45 min at 37 °C. Fluorescence readings were obtained using a VictorTM 1420 Multilabel Counter (PerkinElmer Inc., Waltham, MA, USA) with an excitation wavelength of 355 nm and an emission of 460 nm, or EnVision 2104 Multilabel Reader (PerkinElmer, Waltham, MA, USA) with an excitation wavelength of 370 nm and an emission of 460 nm.

## Figures and Tables

**Table 1:** Fucoidan against SIRT1-SIRT3 deacetylation activities. The data are presented as means ± SD, *n* = 3.

Fucoidan (µg/mL)	Fold Increase in Activity ± SD		
	SIRT1	SIRT2	SIRT3
10	0.96 ± 0.04	0.87 ± 0.06	0.99 ± 0.01
100	0.94 ± 0.02	0.82 ± 0.02	0.98 ± 0.02

## Supplementary Materials

The following are available online at www.mdpi.com/1660-3397/15/6/190/s1, Figure S1: Fractionation (F1–F5) of *F. distichus* on the LC-MS/MS API-5500 and on the HPLC-DAD were collected using an Eclipse XDB-C8 column (9.4 mm × 250 mm, 5 µm). Fractions were collected between 0.1–1.0 min (F1), 1.0–1.3 min (F2), 8.0–8.5 min (F3), 10.0–10.5 min (F4), 11.0–11.4 min (F5). Figure S2: HPLC-MS chromatograph of subfraction F1 (10 µL). The flow rate was 0.6 mL/min a nd the injection volume was 10 μL of 7.5 mg/mL.
