# Supplementary material for: The Identification of a SIRT6 Activator from Brown Algae Fucus distichus
Source: Mar Drugs. 2017 Jun 21;15(6):190. doi: 10.3390/md15060190 (PMC5484140; doi:10.3390/md15060190)
Supplement: Supplementary file 1 [file marinedrugs-15-00190-s001.pdf]

Supplementary Materials: The Identification of a SIRT6 Activator from Brown Algae *Fucus distichus*

Minna K. Rahnasto-Rilla, Padraig McLoughlin, Tomasz Kulikowicz, Maire Doyle, Vilhelm A. Bohr, Maija Lahtela-Kakkonen, Luigi Ferrucci, Maria Hayes and Ruin Moaddel

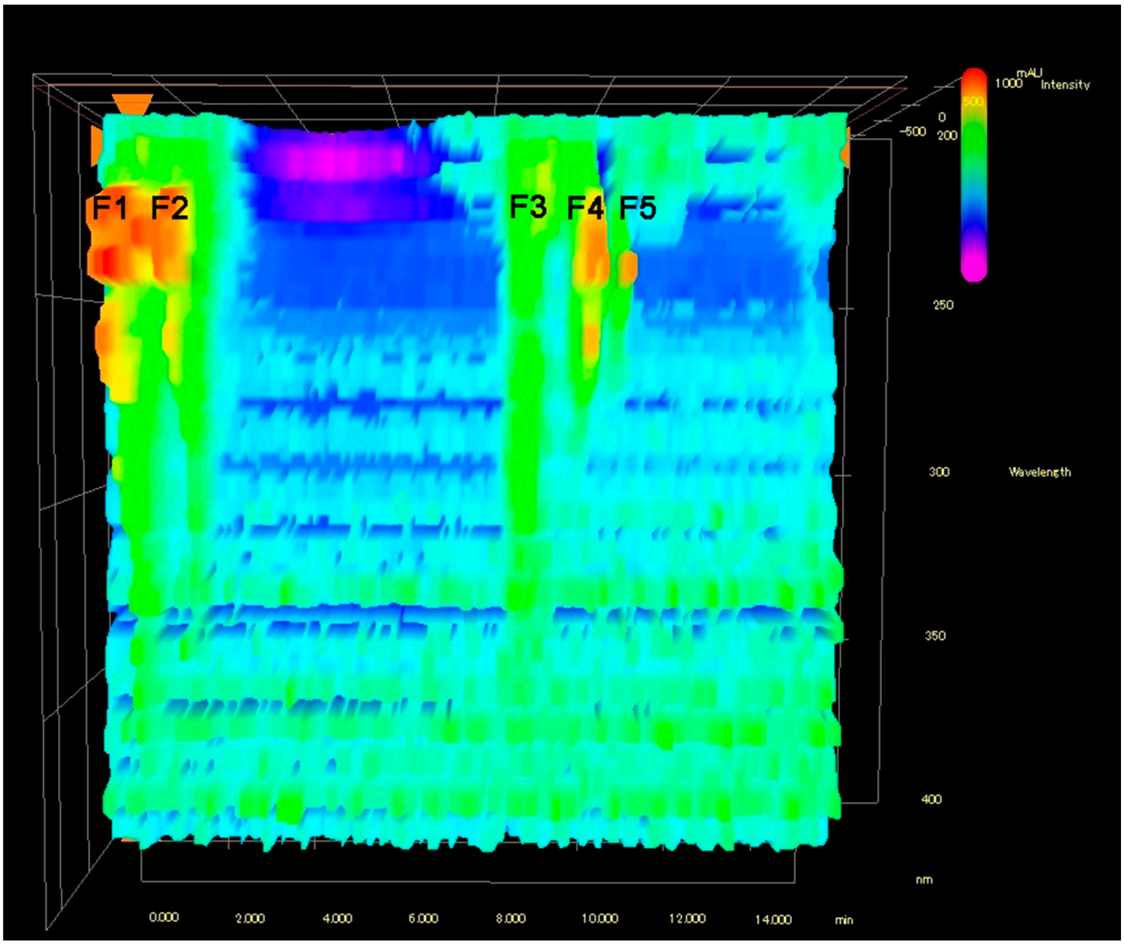

Figure S1. Fractionation (F1–F5) of *F. distichus* on the LC-MS/MS API-5500 and on the HPLC-DAD were collected using an Eclipse XDB-C8 column (9.4 mm × 250 mm, 5 µm). Fractions were collected between 0.1–1.0 min (F1), 1.0–1.3 min (F2), 8.0–8.5 min (F3), 10.0–10.5 min (F4), 11.0–11.4 min (F5).

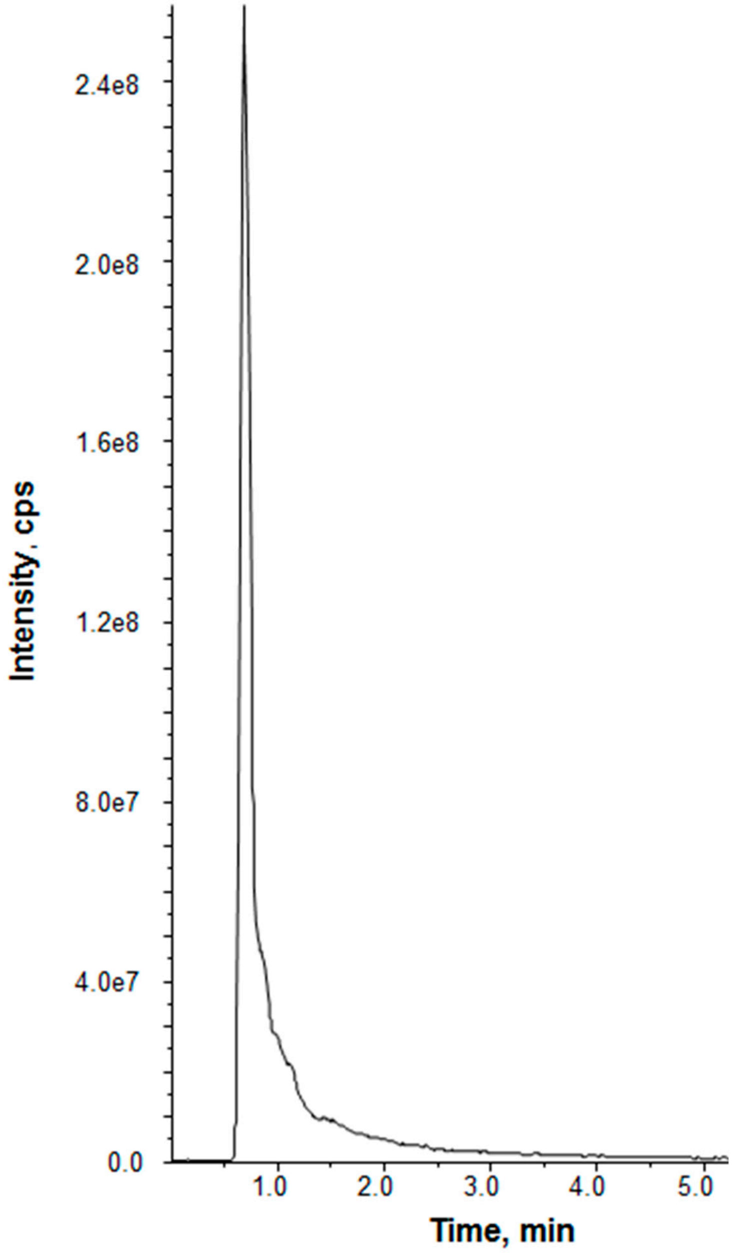

Figure S2. HPLC-MS chromatograph of subfraction F1 (10 μL). The flow rate was 0.6 mL/min and the injection volume was 10 μL of 7.5 mg/mL.
